# Night Owls and Early Birds: The Role of Adolescents' Chronotype on Educational Identity Trajectories

**DOI:** 10.1002/ijop.70108

**Published:** 2025-09-08

**Authors:** Valeria Bacaro, Francesca De Lise, Vincenzo Natale, Lorenzo Tonetti, Elisabetta Crocetti

**Affiliations:** ^1^ Department of Psychology “Renzo Canestrari” University of Bologna Bologna Italy

**Keywords:** adolescence, chronotype, educational identity, longitudinal, sleep

## Abstract

Chronotype is the preference for sleep and activity timing, differentiating individuals into morning (i.e., waking and sleeping early), evening (i.e., preferring later sleep patterns) and intermediate (i.e., falling between these extremes) types. Adolescents' chronotype has been linked to school performance, but its connection to the development of their educational identity has been overlooked. A stable educational identity involves the interplay of three processes: commitment (i.e., identification with educational choices), in‐depth exploration (i.e., exploring and reflecting on commitments) and reconsideration of commitment (i.e., questioning current commitments and seeking alternatives). This longitudinal study investigates whether adolescents' chronotypes can be associated with trajectories of educational identity processes and how the latter may mediate the link between adolescents' chronotypes and school performance. The study followed 1156 adolescents (*M*_age_ = 15.69, SD_age_ = 1.20; 51.65% females) over four assessments spanning 1 year. Multigroup analyses showed that adolescents with an evening chronotype reported lower initial levels of educational commitment and in‐depth exploration, coupled with higher reconsideration of commitment than adolescents with a morning chronotype. Additionally, a mediating effect of in‐depth exploration was found in the link between chronotype and school performance. This study highlights the detrimental role of evening chronotype in educational identity development.

## 1 Introduction

Adolescents face crucial physical, cognitive and emotional changes. Combined with the increasing demands of the social contexts in which they are embedded, these phenomena may lead to dramatic transformations in youth' sleep/wake cycle and patterns (Carskadon 2011). One key aspect of the sleep/wake patterns is chronotype (i.e., the circadian preference for the time of the day best suited for waking and sleeping behaviours). During adolescence, the chronotype typically progressively shifts toward eveningness, as manifested in the delay in going to bed and getting up and in the preference for activities in the evening (Roenneberg et al. 2004; Tonetti et al. 2008). Extensive research has focused on how evening chronotype can be considered a risk factor for several aspects of adolescents' mental health (e.g., anxiety and depression symptoms; for a review, see Cheung et al. 2023), affecting several facets of youth' daily life experiences.

In particular, adolescents spend most of their daily time in school and performing school‐related activities, and previous research showed how having an evening chronotype was associated with lower school performance, especially in adolescents (Tonetti et al. 2015). One of adolescents' pivotal tasks is establishing a clear identity (Erikson 1968) by exploring alternatives and making meaningful commitments in developmentally relevant domains, such as the educational one (Crocetti et al. 2023). Developing a stable sense of educational identity is closely intertwined with adolescents' academic achievement (Pop et al. 2016) and well‐being (De Lise et al. 2024). Nevertheless, the role of chronotype in shaping adolescents' educational identity development and its potential mediating role in the relation between chronotype and school performance remain unexplored. Building on this premise, the present study investigated whether adolescents' chronotype is associated with their developmental trajectories of educational identity and whether the latter mediate the link between chronotype and school performance.

### 1.1 Adolescents' Biological Rhythms: The Role of Chronotype

Chronotype is an influential aspect of human sleep–wake patterns, defined as individuals' preference for earlier or later sleep and activity times within the 24‐h day (Adan et al. 2012). Thus, individuals can be differentiated into those with a *morning chronotype*, also known as ‘early birds’, who have a propensity for morning activities, early awakenings and early bedtimes; an *evening chronotype*, so‐called ‘night owls’, with the tendency to prefer evening activities, late awakenings and late bedtimes; and an *intermediate chronotype*, which identifies individuals who fall within these two extremes. While most individuals report an intermediate chronotype, the preference for the morning and evening chronotypes can change across the lifespan (Natale and Cicogna 2002).

It has been theorised that adolescents' sleep patterns are affected by a ‘perfect storm’ (Carskadon 2011), with biopsychosocial factors impacting their sleep and leading to a progressive shift toward an evening chronotype. Specifically, adolescents experience maturational changes in intrinsic bioregulatory factors, associated with a progressive shift toward eveningness (Roenneberg et al. 2004), even more evident in recent years (Arns et al. 2021). Moreover, chronotype has been found to be associated with pubertal development (Foley et al. 2018), with an evening chronotype more prevalent in more mature adolescents (Hagenauer et al. 2009) and females reaching their peak of eveningness earlier than males (Dìaz Morales et al. 2023). Concurrently, psychosocial factors such as self‐selected bedtimes, academic pressures and the use of technology and social networks in the evening can influence adolescents' sleep/wake cycle, leading to a preference for evening activities and later bedtimes (Becker et al. 2015). This contrasts with societal pressures for an early rise time (i.e., early school start times), forcing adolescents to be awake and perform at a time misaligned with their circadian rhythms.

The prevalence and tendency of reporting an evening chronotype among adolescents is considered a matter of concern, representing a public health issue (Touitou 2013), as it is considered a risk factor for adolescents' adjustment. Specifically, the evening chronotype has been found to be associated with internalising and externalising symptoms (e.g., Merikanto et al. 2017), rumination (more likely to be experienced at the end of the day) and dysfunctional emotion regulation strategies (e.g., Bauducco et al. 2020). These results suggest how chronotype can play a crucial role in establishing a vicious cycle between sleep/wake patterns and adjustment. This interplay between adolescents' chronotype and their adjustment also unfolds in the context in which young people spend most of their time: the school context. Consistent evidence has shown that adolescents' sleep/wake cycle and chronotype are related to their academic performance, with an evening chronotype being associated with poorer grades (for a review, see Tonetti et al. 2015). Thus, it is important to unravel which factors could explain the link between chronotype and school performance. This study tackled this aspect by addressing the role of educational identity.

### 1.2 Educational Identity in Adolescence

The development of the educational identity represents a key task during adolescence (Erikson 1968). Educational identity is particularly relevant for adolescents, considering that during this life stage, they start making significant educational decisions for the first time (Verhoeven et al. 2019). Additionally, it is strongly linked to their future career and professional identity (Crocetti et al. 2025; Marinica and Negru‐Subtirica 2024).

According to the three‐factor identity model (Crocetti et al. 2008), adolescents can develop their educational identity through a dynamic and iterative process based on the interplay among commitment, in‐depth exploration and reconsideration of commitment. Specifically, *commitment* refers to enduring choices individuals have made about their education and the self‐confidence they derive from them. *In‐depth exploration* indicates the extent to which individuals think actively about the educational commitments they have made, reflecting on their choices, searching for additional information and talking with others about their commitments. *Reconsideration of commitment* refers to comparing current educational commitments with possible alternatives because the current ones are no longer satisfactory.

So far, research has highlighted that adolescents who have found meaningful identity commitments that they do not need to reconsider report better psychosocial adjustment levels (for a review, see Branje et al. 2021). Additionally, educational identity was found to be linked with adolescents' multiple dimensions of well‐being (De Lise et al. 2024) and school‐related outcomes, such as academic achievement (Pop et al. 2016). Conversely, individuals' state of health can play an important role in fostering or hindering identity formation processes. Specifically, previous research has highlighted that individuals who report somatic symptoms can experience difficulties in forming and maintaining solid commitments (Raemen et al. 2023). This evidence suggests that when adolescents present some risk factors, they may struggle more in defining their identity. In line with this, it is of utmost importance to understand if adolescents' preferences for different sleep/wake patterns can also represent protective or risk factors for their identity development.

### 1.3 Exploring the Role of Chronotype in Shaping Adolescents' Educational Identity

Building upon the goodness‐of‐fit model (Windle and Lerner 1986), adolescents' chronotypes can be theorised to play an important role in their development. This model emphasises the dynamic interaction between individual characteristics and their environment. Specifically, a positive psychosocial adjustment is promoted when there is a good fit between individuals' characteristics, such as their chronotype, and the demands and opportunities of the social context in which they are embedded, such as school demands. In contrast, a poor fit can lead to stress, maladjustment and developmental difficulties.

In line with these theoretical premises, adolescents' chronotypes may help explain differences in the development of educational identity. Specifically, an evening chronotype can lead to a misalignment between adolescents' sleep/wake rhythms and the demands of their school and social schedules (Crowley et al. 2018; Estevan et al. 2020). In fact, adolescents with an evening chronotype tend to be out of sync with early morning school requirements, performing better during late hours despite being expected to wake up early and be vigilant during the early morning school hours (Preckel et al. 2011). This misalignment often results in sleep deprivation and poor sleep quality, especially during school days (Rodríguez Ferrante et al. 2024).

Previous studies highlighted how short sleep duration, later bedtime and a higher amount of social jet‐lag (i.e., lack of synchrony between biological clocks and social clocks; Wittmann et al. 2006) were associated with poor school performance (Bacaro et al. 2023). Experiencing this discrepancy repeatedly throughout the school year can hinder the development of a strong educational identity. At the same time, adolescents' educational identity is closely linked to their school performance (Pop et al. 2016). Nevertheless, no studies evaluated the connection between adolescents' different chronotypes and their educational identity development, considering also the possible effects on school performance.

## 2 The Current Study

Adolescents' chronotypes are associated with several facets of well‐being. Previous research has emphasised that early school start times and schedules may disrupt adolescents' sleep/wake cycle, especially disadvantaging those with an evening chronotype. So far, available evidence has mainly focused only on academic performance, showing that adolescents with an evening chronotype reported lower academic achievement than those with a morning chronotype (e.g., Goldin et al. 2020). However, it remains unclear whether chronotypes can influence adolescents' ability of coping with developing a solid educational identity and the potential implications for their school performance. Building on these considerations, the present longitudinal study aimed to (a) examine whether adolescents' chronotypes (i.e., morning, intermediate and evening) are related to differences in developmental trajectories of educational identity processes (i.e., commitment, in‐depth exploration and reconsideration of commitment) and (b) investigate if the adolescents' developmental trajectories of educational identity processes can mediate the link between adolescents' chronotype and school performance. It was hypothesised that adolescents with an evening chronotype might have more difficulties establishing a stable sense of identity (i.e., high commitment, high in‐depth exploration and low reconsideration of commitment) over time as their personal preference contrasts with school organisation and demands. Such difficulties would be reflected in increasing identity uncertainty as indicated by low commitment, low in‐depth exploration and high reconsideration of commitment, which, in turn, may negatively affect their school performance.

## 3 Methods

### 3.1 Participants

Participants of this study are drawn from the ongoing longitudinal *ERC‐Consolidator project IDENTITIES “Managing identities in diverse societies: A developmental intergroup perspective with adolescents"*. A total of 1156 adolescents (*M*_age_ = 15.69, SD_age_ = 1.20 at baseline, 51.65% females) from two cohorts attending the 1st (49.44%) and 3rd (50.56%) years of several high schools located in the North‐East of Italy (i.e., Emilia‐Romagna region) were followed for four assessments across 1 year (i.e., T1: January/February 2022, T2: April/May 2022, T3: September/October 2022, T4: January/February 2023). Students were enrolled in different school tracks. Specifically, some attended a university‐oriented track (45.63%), followed by those enrolled in a technical (31.86%) and a vocational (22.51%) school. Regarding the school schedule, most adolescents attended school 6 days per week (except for 79 participants in school divisions with a 5‐day schedule). However, the total school hours varied depending on the school track, with adolescents attending university‐oriented schools having a weekly schedule ranging from 27 to 33 h, those in technical schools attending 32 h per week and those in vocational schools having the most variable schedule, with weekly hours ranging from 32 to 64, depending on the specific programme and specialisation. The school entrance time generally started between 7:50 AM and 9:15 AM, with most students beginning at 8:00 AM. The school exit time varied significantly, ranging from 12:00 to 15:50, with most students finishing at 13:00. These variations reflect the structural differences among school tracks, with university‐oriented schools generally having shorter school days while vocational ones having longer schedules, often including additional laboratory hours or practical training sessions.

Most adolescents participated in all four assessments (53.63%), while almost all (87.11%) completed at least two. The completion rate at the item level was very high (94% at T1, 77.3% at T2, 66.8% at T3 and 59.5% at T4). The Little's (1988) Missing Completely at Random (MCAR) test conducted on the study variables yielded a normed *χ* ^2^ (*χ* ^2^/df = 2175.90/1687) of 1.29, indicating that data were likely missing completely at random. Since 22 participants (1.90%) did not complete the measure regarding their chronotype at T1, they were excluded from further analyses. Therefore, the final sample included 1134 adolescents.

### 3.2 Procedure

The present study was approved by the Ethics Committee of Alma Mater Studiorum University of Bologna (Italy) as part of the IDENTITIES project. Schools were selected through a stratified (by track, level of urbanisation) randomised method, and principals were approached to present the project. Upon their approval, the study was presented to students and their parents, who received written and detailed information about it. Parents' active consent was obtained before their children's participation. Active consent was also obtained from adolescents of age, while their underage peers provided their assent to participate in the project. Participation in the study was voluntary, and students were informed they could withdraw their consent at any time. All participants in each assessment completed an online questionnaire during class hours. Research assistants were present in the class to answer possible questions from students. Participants were required to create a personal code to ensure confidentiality and pair their answers over time.

### 3.3 Measures

#### 3.3.1 Demographics

Participants' sociodemographic information (e.g., age, biological sex) was collected at baseline (T1).

#### 3.3.2 Chronotype

The chronotype of adolescents was assessed with the Italian version (Tonetti et al. 2006) of the reduced Morningness‐Eveningness Questionnaire for Children and Adolescents (rMEQ‐CA), which was also validated through actigraphy (Tonetti et al. 2024). The instrument consists of five items, each with a different response scale. However, all items are scored so that higher values correspond to a greater morningness tendency. Specifically, participants were requested to indicate their ideal get‐up time (i.e., from 1 ‘after 10:46 AM’ to 5 ‘before 6:30 AM’), their usual bedtime (i.e., from 1 ‘after 2:16 AM’ to 5 ‘before 9 PM’), at what time of the day their ‘best peak’ is reached (i.e., from 1 ‘between 8 PM and 03:59 AM’ to 5 ‘between 4 AM and 7:59 AM’), the tiredness after the morning awakening (i.e., from 1 ‘very tired’ to 4 ‘very awake’) and which type of people (‘morning’ or ‘evening’ type) they consider themselves to be (i.e., from 0 ‘definitely an evening type’ to 6 ‘definitely a morning type’). The total score, obtained by summing up scores assigned to each question, ranges from 4 to 25. Individuals with a morning chronotype are those with a total score between 19 and 25, intermediate between 11 and 18 and evening between 4 and 10 (Tonetti et al. 2006). Adolescents completed this questionnaire at baseline (T1). Most of the participants reported an intermediate chronotype (73%), while the remaining adolescents reported an evening chronotype (19.6%), and only a small part showed a morning chronotype (7.4%), as typically documented in this age population (e.g., Saxvig et al. 2021).

#### 3.3.3 Educational Identity Processes

Commitment, in‐depth exploration and reconsideration of commitment were measured using the Italian version (Crocetti et al. 2010) of the Utrecht‐Management of Identity Commitments Scale (U‐MICS, Crocetti et al. 2008). The instrument consists of 13 items scored on a 5‐point Likert‐type rating scale, ranging from 1 (completely false) to 5 (completely true). Sample items include: ‘My education gives me certainty in life’ (commitment; 5 items), ‘I think a lot about my education’ (in‐depth exploration; 5 items) and ‘I often think it would be better to try to find a different education’ (reconsideration of commitment; 3 items). Adolescents completed this measure at all the four time points of the study. Cronbach's Alphas are reported in Table S1.

#### 3.3.4 School Performance

Adolescents' school performance was assessed by asking them to self‐report their grade‐point average (GPA). The Italian grading system is based on a 10‐point scale, where 1 is the lowest possible grade and 10 is the highest. A grade of at least six is required to achieve sufficiency. Adolescents reported this information at the last time point (T4).

### 3.4 Statistical Analyses

#### 3.4.1 Preliminary Analyses

First, descriptive analyses and correlations between study variables were estimated. Then, a one‐way analysis of variance (ANOVA) was conducted to test for significant differences in school performance (i.e., GPA) between the three chronotype groups (i.e., morning, intermediate and evening). The HSD Tukey post hoc test was used for pairwise comparisons. Additionally, differences in adolescents' chronotype distribution and identity processes over time based on the different school track characteristics were tested. Specifically, a chi‐square test was performed to analyse differences in the distribution of adolescents' chronotypes across the adolescents' school tracks. Then, a repeated‐measures ANOVA was conducted to examine differences in identity processes over time based on the adolescents' school tracks. This set of analyses was conducted using IBM SPSS Version 28.0. Finally, longitudinal measurement invariance was examined for the three identity processes using M*plus* 8.11 (Muthén and Muthén 2017) using the Maximum Likelihood Robust (MLR) estimator (Satorra and Bentler 2001). Longitudinal measurement invariance allows for determining whether the same measure can be used to reliably assess latent constructs over time. Establishing measurement invariance involves conducting a series of increasingly constrained structural equation models and testing whether the differences between them are statistically significant and substantial (van de Schoot et al. 2012). Specifically, first, the configural model, which was used as the baseline model, was tested. The configural model implies that the same factorial structure holds across time. Then, it was compared to the metric model, in which factor loadings were constrained to be equal across time, implying that measures maintain the same meaning and contribute to the latent factor equally over time. If metric invariance was reached, this model was tested against the scalar model, in which the intercepts are also equal across time points. Multiple indices were used to evaluate the model fit (Byrne 2012): the Comparative Fit Index (CFI) with values higher than 0.90 representing an acceptable fit and values higher than 0.95 displaying an excellent fit; the Standardised Root Mean Square Residual (SRMR) and the Root Mean Square Error of Approximation (RMSEA), with values less than 0.08 indicative of an acceptable fit and values less than 0.05 indicating excellent fit (Byrne 2012); and 90% Confidence Interval for the RMSEA, with the upper bound lower than 0.10 representing an acceptable model fit (Chen et al. 2008). For model comparison purposes, if at least two of the following criteria are satisfied, measurement invariance could be established: (a) a non‐significant Δ*χ*_SB_^2^, (b) ΔCFI < −0.010 and (c) ΔRMSEA < 0.015 (Chen 2007; Satorra and Bentler 2001).

#### 3.4.2 Main Analyses

All the main analyses were conducted in M*plus* 8.11 (Muthén and Muthén 2017), with the MLR estimator (Satorra and Bentler 2001). In order to address the first aim of this study, to capture individual differences in continuous trajectories over time, multivariate Latent Growth Curve Models (LGCM; Duncan et al. 1999) were estimated for the three processes of educational identity. Using these models allows us to model individual growth trajectories, accounting for measurement error, and reflecting accurate developmental patterns (Duncan and Duncan 2009). LGCM expresses growth in each variable as an intercept (i.e., initial level) and slope (i.e., rate of change). To capture the best fitting model, different types of models were estimated: an intercept‐only model, assuming no change over time; a linear model, assuming a linear change over time; and a free‐change model, assuming possible non‐linear growth by using free slope factor estimation obtained by fixing two slope factor loadings for model identification and freely estimating the others (Muthen and Khoo 1998). The fit of each model was examined following the same procedure described above and differences between intercept‐only, linear and free‐change models were identified if at least two of the following criteria were met: a Δ*χ*_SB_^2^ significant at *p* < 0.05 (Satorra and Bentler 2001), ΔCFI ≥ −0.010, and ΔRMSEA ≥ 0.015 (Chen 2007). A multigroup approach with multivariate LGCM analyses was applied to test the hypotheses of this study. Specifically, whether intercept and slope values of the three educational identity processes significantly differ depending on the participants' chronotype at T1 (determined by their scores on the rMEQ‐CA scale) was tested. The Wald test was applied to conduct pairwise comparisons.

In order to address the second aim of this study, a mediation model was tested in which chronotype was dummy coded (with the evening group as the reference category) and treated as the independent variable, while the latent trajectories of educational identity processes were the mediators, and GPA was the dependent variable. Mediating effects were examined using the indirect command procedure available in M*plus*. Data, analysis codes and outputs can be retrivied from https://osf.io/4fhqx/.

## 4 Results

### 4.1 Preliminary Analyses

Cronbach's alphas, means, standard deviations and correlations among study variables are reported in Table S1. The ANOVA to investigate differences in school performance based on adolescents' chronotype yielded a significant effect (*F* (2) = 3.83, *p* = 0.022, *η* ^2^ = 0.011), with Tukey post hoc comparisons highlighting that adolescents with an evening chronotype reported significantly lower GPA at T4 than those with a morning chronotype, while adolescents with an intermediate chronotype did not differ from the other two groups (see Table S2). The chi‐square test results, which examined differences in the distribution of adolescents' chronotypes across the school tracks, showed no significant differences (see Table S3). Finally, the results of the repeated‐measures ANOVA to examine changes in the three identity processes over time across adolescents' school tracks showed no significant differences (*F* (3, 1150) = 2.02, *p* = 0.060, *η* ^2^ = 0.010). All levels (i.e., configural, metric and scalar) of longitudinal measurement invariance of educational identity processes were established (see Table S4).

### 4.2 Educational Identity Trajectories for All Sample

The linear model for the three educational identity processes was the best fitting model (*χ* ^2^ = 310.812, df = 51, CFI = 0.932, TLI = 0.912, RMSEA = 0.062 [0.055, 0.069]; see Table S5). As can be seen from Table 1, levels of all three processes were medium‐high, with intercepts ranging from 3.02 to 3.12 on a scale from 1 to 5. Additionally, results showed that for each process, there were non‐significant negative slopes with significant variability, indicating that, in line with the aims of the study, diverse developmental trajectories could be differentiated within the general sample.

**TABLE 1:** Descriptive statistics of the linear model for the three identity processes.

	Intercepts		Slopes	
Educational identity	*M* (SE)	*σ* ^2^ (SE)	*M* (SE)	*σ* ^2^ (SE)
Commitment	3.121*** (0.023)	0.501*** (0.032)	−0.016 (0.009)	0.036*** (0.007)
In‐depth exploration	3.123*** (0.019)	0.312*** (0.024)	−0.007 (0.008)	0.023*** (0.006)
Reconsideration of commitment	3.018*** (0.024)	0.470*** (0.037)	−0.017 (0.010)	0.036*** (0.009)

*Note: σ* ^2^ variance. *N* = 1134.

Abbreviations: *M* = mean, SE = standard error.

*** *p* < 0.001.

### 4.3 Educational Identity Trajectories Based on the Adolescents' Chronotype at Baseline

**FIGURE 1:**
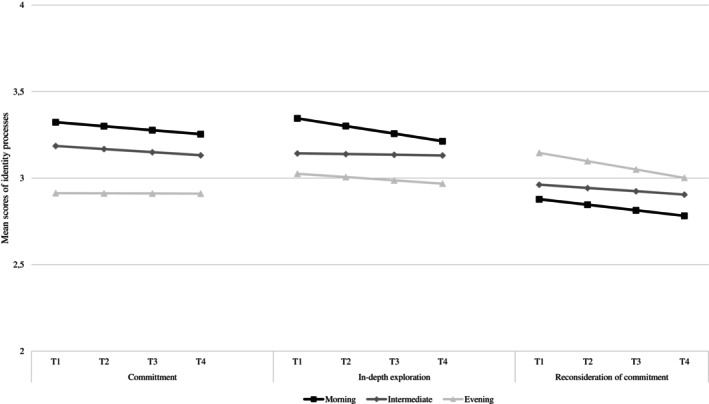
Latent trajectories for the three educational identity processes based on adolescents' chronotype. For the sake of clarity, the figure displays a range from 2 to 4 to enhance visibility, but the original scale ranges from 1 to 5. *N* = 1134.

To address the first aim, LGCM was tested to investigate whether adolescents reported different educational identity trajectories based on their chronotype (i.e., morning, intermediate and evening). Results (see Table 2 and Figure 1) showed that the main differences between the three groups based on chronotype were related to the initial levels (i.e., intercepts) of the three educational identity processes, while no significant differences between the three groups were highlighted in their rates of change (i.e., slopes). Specifically, the initial levels of commitment were significantly lower in adolescents with an evening chronotype compared to those with an intermediate (Wald = 14.547, *p* < 0.01) and a morning one (Wald = 16.740, *p* < 0.001). No significant differences were highlighted between adolescents with intermediate and morning chronotypes in the commitment process. Further, adolescents with a morning chronotype reported significantly higher initial levels of in‐depth exploration compared to those with intermediate (Wald = 7.228, *p* < 0.01) and evening (Wald = 13.189, *p* < 0.01) chronotypes. Additionally, adolescents with an intermediate chronotype reported higher levels of in‐depth exploration compared to those with an evening one (Wald = 4.453, *p* < 0.05). Finally, concerning the reconsideration of commitment process, adolescents with an evening chronotype showed significantly higher initial levels compared to the ones with morning (Wald = 5.973, *p* < 0.05) and intermediate chronotypes (Wald = 7.479, *p* < 0.01). No significant differences were highlighted between adolescents with morning and intermediate chronotypes in the reconsideration of commitment. Nevertheless, adolescents with an evening chronotype showed a significant decrease in the reconsideration of commitment process over time.

**TABLE 2:** Descriptive statistics of the identity trajectories based on the adolescents' chronotype at baseline (T1).

	Intercepts		Slopes	
	*M* (SE)	*σ* ^2^ (SE)	*M* (SE)	*σ* ^2^ (SE)
Commitment				
Morning chronotype	3.323*** (0.089)b	0.475*** (0.010)	−0.023 (0.033)	0.010 (0.019)
Intermediate chronotype	3.186*** (0.027)^b^	0.466*** (0.037)	−0.018 (0.011)	0.040*** (0.008)
Evening chronotype	2.913*** (0.061)^a^	0.544*** (0.079)	−0.001 (0.024)	0.023 (0.018)
In‐depth exploration				
Morning chronotype	3.345*** (0.072)^c^	0.319*** (0.092)	−0.044 (0.033)	0.034 (0.021)
Intermediate chronotype	3.143*** (0.022)^b^	0.290*** (0.026)	−0.004 (0.009)	0.022** (0.007)
Evening chronotype	3.025*** (0.051)^a^	0.340*** (0.061)	−0.019 (0.021)	0.012 (0.013)
Reconsideration of commitment				
Morning chronotype	2.878*** (0.091)^a^	0.400*** (0.100)	−0.032 (0.043)	0.053* (0.027)
Intermediate chronotype	2.963*** (0.029)^a^	0.459*** (0.043)	−0.019 (0.012)	0.036** (0.011)
Evening chronotype	3.146*** (0.061)^b^	0.493*** (0.099)	−0.048* (0.024)	0.031 (0.025)

*Note: σ* ^2^ variance. Means with different subscripts differ significantly at the Wald test (*p* < 0.05). *n*_morning_ = 84, *n*_intermediate_ = 828, *n*_evening_ = 222.

Abbreviations: *M* = mean, SE = standard error.

* *p* < 0.05.

** *p* < 0.01.

*** *p* < 0.001.

### 4.4 Mediating Role of Educational Identity Trajectories in the Link Between Chronotype and School Performance

**FIGURE 2:**
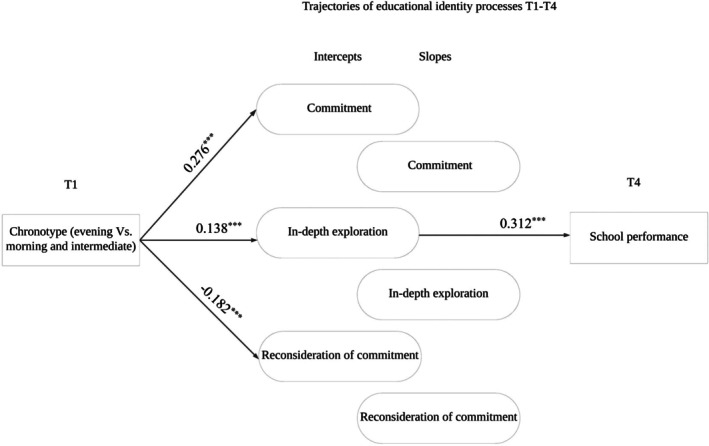
Mediating model of educational identity trajectories in the link between chronotype and school performance. For sake of clarity, only significant paths are reported. *N* = 1134; ***p < 0.001.

In line with the second aim of this study, mediating effects were examined to investigate if the adolescents' developmental trajectories of educational identity processes can explain the link between adolescents' chronotype and school performance. A significant indirect effect was found (see Figure 2). Specifically, having a morning or intermediate chronotype compared to the evening one was associated with higher levels of school performance via the intercept of in‐depth exploration (standardised indirect effect = 0.02 [0.01, 2.09], *p* = 0.037).

## 5 Discussion

Adolescents tend to shift to an evening chronotype that is intricately linked with their adjustment (for a review, see Bauducco et al. 2020) and with different aspects of daily life experiences. Adolescents with an evening chronotype have been found to be disadvantaged in the school context, considering that their sleep/wake rhythms are not aligned with the demands of being productive and vigilant during the early morning (Preckel et al. 2011). Nevertheless, previous research primarily focused on the effect of chronotype on academic performance, leaving unexplored its link with their educational identity processes. This study aimed to fill this gap by tackling the association between adolescents' different chronotypes (i.e., morning, intermediate and evening) and their educational identity processes (i.e., commitment, in‐depth exploration, reconsideration of commitment) trajectories over time and the potential mediating role of these trajectories in the link between chronotype and school performance.

### 5.1 Chronotypes in Adolescence: A Hidden Key for Understanding Educational Identity

This study highlighted that adolescents with different chronotypes reported variations in educational identity processes, especially in their initial levels. Adolescents with an evening chronotype reported a more unstable sense of educational identity compared to those with a morning one. That is, adolescents with an evening chronotype reported significantly lower initial levels of commitment and in‐depth exploration and higher levels of reconsideration of commitment than their peers with a morning chronotype.

This expands previous evidence related to the school context that has documented how adolescents with evening chronotype may be disadvantaged in the school context, perhaps due to the effects of sleep deprivation resulting from the mismatch between their chronotype and the school demands (for review, see Tonetti et al. 2015). Previous findings also highlighted how this negative effect of chronotype seems to be persistent even when school starts later (Rodríguez Ferrante et al. 2023), indicating how chronotype may affect academic success through other mechanisms. Considering the importance of the alignment between individual characteristics and environmental demands, as emphasised in the goodness‐of‐fit model (Windle and Lerner 1986), the results of this study provide valuable insight into how adolescents with an evening chronotype adjust to school setting. Specifically, the current study may help clarify whether the disadvantages related to having an evening chronotype stem from endogenous characteristics or if external factors, such as school‐related experiences, exacerbate these challenges. The results of this study highlighted how adolescents with an evening chronotype struggle more to align with the demands and structure of the school context and that this misalignment extends beyond academic performances, hindering their overall school experience.

Additionally, when evaluating the mediating role of educational identity process trajectories in the link between chronotype and academic performance, results showed that the intercept in‐depth exploration mediated the relation between chronotype and later academic performance. Specifically, compared to those with evening one, adolescents with a morning or intermediate chronotype reported higher academic performance with in‐depth exploration acting as a mediator. This result aligns with the previous conceptualisation of in‐depth exploration as a double‐edged sword linked to both positive and negative outcomes (Crocetti et al. 2008). Since in‐depth exploration is a cognitively demanding process requiring adolescents to actively reflect on and evaluate the meaning of their educational commitments, it likely depends on the engagement of specific cognitive strategies (Negru‐Subtirica et al. 2017). This result expands prior research (Bacaro et al. 2023), highlighting that adolescents experiencing sleep debt, common among those with an evening chronotype, may lack the cognitive resources needed to compensate for sleep‐related deficits. As a result, they may struggle to actively engage in in‐depth exploration of their educational commitments, ultimately placing them at a disadvantage in terms of academic performance.

These findings contribute to bridging the growing lines of research that look at how chronotype and identity consolidation, respectively, matter for adolescents' psychosocial adjustment. Indeed, adolescents with an evening chronotype are considered more at risk for a range of adverse health outcomes compared to those with a morning one (Cheung et al. 2023). Similarly, previous research highlighted the positive relation between having a well‐defined educational identity and experiencing better health‐related outcomes (e.g., De Lise et al. 2024). This study takes a further step toward the integration of these research streams by showing how evening chronotype can contribute to a lack of a coherent sense of educational identity, which can, in turn, result in a negative spiral. Thus, the evening chronotype, linked to a less stable educational identity and negative academic outcomes, appears as a significant risk factor for adolescents' psychosocial adjustment.

### 5.2 Practical Implications

The results of this study have important practical implications, especially for the school context. They expand existing literature by further elucidating the potential advantages of postponing school start times during adolescence, in line with the American Academy of Pediatrics recommendations (Adolescent Sleep Working Group 2014). In addition to this, and in line with the theoretical premises of this study, it is also of utmost importance to consider individual characteristics and needs. For instance, distance from home to school implies that some adolescents may need to wake up very early to be at school on time. This calls for actions to adapt school entrance time to the specific needs of the context in which adolescents are embedded. In this way, it would be possible to reduce the potential effect of external factors, such as school entry schedules, in exacerbating the well‐being of adolescents with an evening chronotype. Thinking about solutions tailored on the specific needs of the students' community may have important implications not only on their cognitive performance but also on other crucial aspects of adolescents' school experience, such as their educational identity, as the present research suggested.

The results of this study also highlighted that the main differences in the educational identity processes between the three chronotypes were tackled at the initial levels but not in their changes over time. This holds significant importance, given that early identification of adolescents with an evening chronotype can be crucial for the implementation of preventive and targeted public health intervention programmes (Cooper et al. 2023). Specifically, within the school context, psychologists, educators and healthcare professionals could first collaborate to map individual differences in chronotype and sleep/wake patterns among youth. This would allow the planning and implementation of targeted interventions enhancing sleep hygiene (i.e., self‐regulatory practices that optimise physiological, behavioural and emotional preparedness for sleep) in at‐risk adolescents, aiming to mitigate the potentially detrimental effect of circadian misalignment (Rodríguez Ferrante et al. 2022). Additionally, at the family level, families should receive guidance on fostering healthy sleep routines (e.g., facilitating regular bedtimes and limiting electronic devices during the evening). Through this combined effort, the crucial role of chronotype can be acknowledged across different ecological contexts of adolescents, helping educators, parents and practitioners to intervene early in preventing the adverse outcomes of circadian misalignment and better supporting adolescents in achieving stable educational identity.

### 5.3 Limitations and Suggestions for Future Research

The present study should be considered in light of some limitations that can suggest directions for future research. First, in this study, adolescents' chronotype was evaluated only at the first time point. While this approach is crucial for identifying individuals with an evening chronotype, that can be an at‐risk group who may require attention and support for early intervention, it does not allow for an assessment of chronotype shifts over time. Previous findings (e.g., Karan et al. 2021; Rodríguez Ferrante et al. 2022) suggested non‐linear trajectories of chronotype through adolescence, with individual differences showing moderate stability. Given the extent of developmental changes in chronotype during adolescence, considering individual differences in the trajectory toward morningness or eveningness and their implications for educational identity processes trajectories would enhance the theoretical understanding of this phenomenon and potential practical implications for preventive interventions.

Second, this study focused on how educational identity processes unfold over time in adolescents with different chronotypes. Further research is needed to tackle whether, on the other side, changes in adolescents' educational identity can also predict changes in their chronotype. In this regard, previous research pointed out a positive loop between identity and well‐being, suggesting that adolescents' solid identity can function as a promotive factor for positive adjustment, and, at the same time, adolescents' well‐being is a prerequisite to successfully accomplishing this pivotal developmental task (De Lise et al. 2024).

Third, this study investigated the role of adolescents' chronotype in the school context, focusing on educational identity development. However, it is essential to acknowledge that other facets of the school context may also be linked with adolescents' sleep/wake cycle and identity. Thus, future research endeavours should incorporate additional dimensions of the school context that could potentially play a role, such as school belonging, engagement and motivation (for a review, see Verhoeven et al. 2019).

Finally, at the first time point of the study, data were collected during one of the peaks of the COVID‐19 pandemic. During that period, several containment measures were applied in schools (e.g., distance learning for positive cases). As a result, although adolescents' chronotype was considered related to their subjective preference, adolescents' sleep/wake patterns could be altered (e.g., Bruni et al. 2022), influencing their perception and educational identity could be affected by differences in teaching and learning processes.

## 6 Conclusion

Evening chronotype during adolescence can represent a risk factor for positive adjustment. Focusing on the school context as one of the most pervasive in adolescents' lives, previous research mainly emphasised the role of adolescents' chronotype for their academic achievement, leading to a gap in understanding its role for other crucial aspects of their school experience, such as the development of a stable sense of educational identity. This study addressed this gap, emphasising the potentially detrimental role of having an evening chronotype for adolescents' educational identity and shedding light on the mediating role of the latter in the link between chronotype and their school performance. Specifically, adolescents with an evening chronotype often report poor school performance, which can lead to disengagement from school commitments and hamper the formation of a stable educational identity, resulting in a negative spiral. Further investigation is needed to disentangle the development and fluctuations of chronotypes during adolescence and their connection to educational identity over time. Such endeavours will enhance efforts aimed at translating these insights into effective prevention and intervention strategies targeting adolescents.

## Author Contributions

**Valeria Bacaro:** Conceptualization (supporting); Formal analysis (lead); Investigation (equal); Methodology (equal); Writing – original draft (lead); Writing – review & editing (lead). **Francesca De Lise:** Conceptualization (supporting); Formal analysis (supporting); Investigation (equal); Methodology (equal); Writing – original draft (equal); Writing – review & editing (supporting). **Vincenzo Natale:** Writing – original draft (supporting); Writing – review & editing (equal). **Lorenzo Tonetti:** Writing – original draft (supporting); Writing – review & editing (equal). **Elisabetta Crocetti:** Conceptualization (lead); Formal analysis (equal); Methodology (equal); Supervision (lead); Project administration (lead); Funding acquisition (lead); Writing – original draft (equal); Writing – review & editing (supporting).

## Ethics Statement

All procedures performed in this study involving human participants were in accordance with the ethical standards of the Ethics Committee of the Alma Mater Studiorum University of Bologna (Italy) and with the 1964 Helsinki declaration and its later amendments or comparable ethical standards.

## Consent

Informed active consent was obtained from participants' parents and assent from the participants themselves was also collected.

## Conflicts of Interest

The authors declare no conflicts of interest.

## Supporting information


**Data S1:** Supporting Information.

## Data Availability

The data, analytic codes, and materials necessary to reproduce the analyses presented in this paper are publicly available on Open Science Framework at the following link: https://osf.io/4fhqx.
