# Supplementary material for: Night Owls and Early Birds: The Role of Adolescents' Chronotype on Educational Identity Trajectories
Source: Int J Psychol. 2025 Sep 8;60(5):e70108. doi: 10.1002/ijop.70108 (PMC12417928; doi:10.1002/ijop.70108)
Supplement: Supplementary file 1 — Data S1: Supporting Information. [file IJOP-60-e70108-s001.docx]

**SUPPLEMENTARY MATERIALS FOR:**

Night Owls and Early Birds: The Role of Adolescents’ Chronotype on Educational Identity Trajectories

**Table S1***.* Cronbach’s alphas, means, standard deviations, and correlations among study variables

**Table S2.** Differences in school performance based on adolescents’ chronotypes

**Table S3.** Distribution of adolescents’ chronotypes across different school tracks

**Table S4.** Longitudinal measurement invariance

**Table S5.** Model fit indices and model comparison

**Table S1.** Cronbach’s alphas, means, standard deviations, and correlations among study variables

|  | *α* | *M* | *SD* | 1. | 2. | 3. | 4. | 5. | 6. | 7. | 8. | 9. | 10. | 11. | 12. |  |
| --- | --- | --- | --- | --- | --- | --- | --- | --- | --- | --- | --- | --- | --- | --- | --- | --- |
| 1. Com T1 | .89 | 3.14 | 0.83 | 1 |  |  |  |  |  |  |  |  |  |  |  |  |
| 2. Exp T1 | .74 | 3.12 | 0.68 | .58^***^ | 1 |  |  |  |  |  |  |  |  |  |  |  |
| 3. Rec T1 | .77 | 2.96 | 0.88 | -.30^***^ | -.02 | 1 |  |  |  |  |  |  |  |  |  |  |
| 4. Com T2 | .90 | 3.17 | 0.82 | .64^***^ | .38^***^ | -.22^***^ | 1 |  |  |  |  |  |  |  |  |  |
| 5. Exp T2 | .77 | 3.17 | 0.70 | .39^***^ | .58^***^ | -.05 | .52^***^ | 1 |  |  |  |  |  |  |  |  |
| 6. Rec T2 | .80 | 3.01 | 0.88 | -.21^***^ | -.07^*^ | .57^***^ | -.24^***^ | -.01 | 1 |  |  |  |  | . |  |  |
| 7. Com T3 | .91 | 3.17 | 0.83 | .60^***^ | .37^***^ | -.28^***^ | .69^***^ | .44^***^ | -.29^***^ | 1 |  |  |  |  |  |  |
| 8. Exp T3 | .78 | 3.16 | 0.71 | .39^***^ | .51^***^ | -.11^**^ | .41^***^ | .61^***^ | -.16^***^ | .63^***^ | 1 |  |  |  |  |  |
| 9. Rec T3 | .82 | 2.91 | 0.88 | -.26^***^ | -.12^***^ | .49^***^ | -.28^***^ | -.11^**^ | .58^***^ | -.27^***^ | -.06 | 1 |  |  |  |  |
| 10. Com T4 | .93 | 3.12 | 0.85 | .53^***^ | .32^***^ | -.28^***^ | .60^***^ | .33^***^ | -.33^***^ | .72^***^ | .47^***^ | -.34^***^ | 1 |  |  |  |
| 11. Exp T4 | .76 | 3.11 | 0.68 | .35^***^ | .46^***^ | -.09^*^ | .34^***^ | .54^***^ | -.12^**^ | .47^***^ | .61^***^ | -.13^***^ | .59^***^ | 1 |  |  |
| 12. Rec T4 | .82 | 2.85 | 0.91 | -.24^***^ | -.12^**^ | .47^***^ | -.28^***^ | -.10^*^ | .57^***^ | -.27^***^ | -.07 | .63^***^ | -.31^***^ | -.01 | 1 |  |
| 13. School Performance T4 |  | 7.22 | 0.88 | .20^***^ | .21^***^ | -.15^***^ | .16^***^ | .14^***^ | -.13^***^ | .24^***^ | .20^***^ | -.13^***^ | .24^***^ | .18^***^ | -.19^***^ | 1 |

***Note.*** T = Time; *α =*Cronbach’s alphas*; M* = Mean; *SD*: Standard Deviation; Com = Educational commitment; Exp = Educational in-depth exploration; Rec = Educational reconsideration of commitment. ^*^ *p* < .05; ^*^ *p* < .01^*^; ^***^ *p* < .001.

**Table S2.** Differences in school performance based on adolescents’ chronotypes

|  | GPA  *M* (*SD*) |
| --- | --- |
| Chronotype |  |
| Morning | 7.39 (0.89) ^a^ |
| Intermediate | 7.32 (0.84) ^a,b^ |
| Evening | 7.10 (0.85) ^b^ |

*Note. M* = Mean; *SD* = Standard Deviation; Means with different subscripts differ significantly at the Tukey post-hoc test (*p* < .05).

**Table S3.** Distribution of adolescents’ chronotypes across different school tracks

|  | Chronotype |  |  |
| --- | --- | --- | --- |
|  | Morning  *n* (%) | Intermediate  *n* (%) | Evening  *n* (%) |
| School track |  |  |  |
| University-oriented | 36 (42.86%) | 396 (47.83%) | 90 (40.54%) |
| Technical | 32 (38.10%) | 259 (31.28%) | 73 (32.88%) |
| Vocational | 16 (19.04%) | 173 (20.89%) | 59 (26.58%) |
| Total | 84 (100%) | 828 (100%) | 222 (100%) |

**Table S4.** Longitudinal measurement invariance

| Educational identity processes | Model fit | | | | | |  | Model comparisons | | | | |
| --- | --- | --- | --- | --- | --- | --- | --- | --- | --- | --- | --- | --- |
|  | χ_SB_^2^ | df | CFI | TLI | SRMR | RMSEA [90% CI] |  | Models | Δχ_SB_^2^(Δdf) | ΔCFI | ΔRMSEA |  |
| Configural invariance (M1) | 2627.286 | 1130 | .939 | .929 | .047 | .032 [.030, .033] |  |  |  |  |  |  |
| Metric invariance (M2) | 2675.821 | 1160 | .938 | .930 | .048 | .031 [.030, .033] |  | M2-M1 | 45.936 (30)^*^ | -.001 | -.001 |  |
| Scalar invariance (M3) | 2948.673 | 1199 | .929 | .921 | .049 | .033 [.032, .035] |  | M3-M2 | 299.529 (39)^***^ | -.009 | -.001 |  |

***Note*.** χ_SB_^2^ = Satorra-Bentler scaled chi-square; df = degree of freedom; CFI = Comparative Fit Index; TLI = Tucker-Lewis Index; SRMR = Standardized Root Mean Square Residual; RMSEA = Root Mean Square Error of Approximation; CI = confidence interval; Δ = change in the parameter.

^*^ *p* < .05; ^***^ *p* < .001

**Table S5.** Model fit indices and model comparison

| Educational identity processes | Model fit | | | | | |  | Model comparisons | | | | |
| --- | --- | --- | --- | --- | --- | --- | --- | --- | --- | --- | --- | --- |
|  | χ_SB_^2^ | df | CFI | TLI | SRMR | RMSEA [90% CI] |  | Models | Δχ_SB_^2^(Δdf) | ΔCFI | ΔRMSEA |  |
| Intercept-only model (M1) | 676.112 | 69 | .840 | .847 | .061 | .081 [.076, .087] |  |  |  |  |  |  |
| Linear model (M2) | **310.812** | **51** | **.932** | **.912** | **.036** | **.062 [.055, .069]** |  | M2-M1 | 366.505 (18)^***^ | -.092 | .019 |  |
| Free-change model (M3) | 321.205 | 48 | .928 | .901 | .032 | .065 [.059, .072] |  | M3-M2 | 5.626 (3)^***^ | .004 | -.003 |  |

***Note*.** χ_SB_^2^ = Satorra-Bentler scaled chi-square; df = degree of freedom; CFI = Comparative Fit Index; TLI = Tucker-Lewis Index; SRMR = Standardized Root Mean Square Residual; RMSEA = Root Mean Square Error of Approximation; CI = confidence interval; Δ = change in the parameter.

^***^ *p* < .001. The best fitting model is indicated in bold.
